# Retinoblastoma in a Young Nigerian Girl: A Case Report From ECWA Eye Hospital, Kano

**DOI:** 10.1155/crop/1733830

**Published:** 2025-06-08

**Authors:** Emamoke Atima-Ayeni, Ayodele Jacob Orugun, Ugbede Idakwo, Oyeronke Komolafe, Mayor Orezime Atima, Akinfenwa Taoheed Atanda, Waziri Garba Dahiru, Sani Kamarudeen Owolabi, Eisuke Shimizu, Nakayama Shintaro, Emmanuel Oluwadare Balogun, Emeka John Dingwoke

**Affiliations:** ^1^ECWA Eye Hospital, Kano, Kano State, Nigeria; ^2^Department of Pathology, Aminu Kano Teaching Hospital, Kano, Kano State, Nigeria; ^3^Department of Pathology, Ahmadu Bello University Teaching Hospital, Zaria, Kaduna State, Nigeria; ^4^Department of Ophthalmology, Keio University School of Medicine, Tokyo, Japan; ^5^Department of Biochemistry, Faculty of Life Sciences, Ahmadu Bello University, Zaria, Kaduna State, Nigeria; ^6^UNESCO International Center for Biotechnology, Nsukka, Enugu State, Nigeria

**Keywords:** chemotherapy, ECWA Eye Hospital, exenteration, fungating ocular mass, histological analysis, Nigeria, pediatric intraocular malignancy, proptosis, retinoblastoma

## Abstract

**Objective:** This report details the case of a 9-year-old Nigerian girl presenting with proptosis and a fungating ocular mass, which was histologically confirmed as retinoblastoma following exenteration.

**Introduction:** Retinoblastoma is the most common pediatric intraocular malignancy, predominantly affecting infants and children under the age of 5, with leukocoria being the most frequent presenting symptom. The occurrence of retinoblastoma in older children is rare and often associated with atypical presentations.

**Case Summary:** A 9-year-old Nigerian girl presented with a 1-year history of progressive left eye symptoms, including redness, pain, decreased vision, and proptosis. Examination revealed a large, fungating ocular mass with no light perception. Imaging studies (ultrasound B-scan and CT scan) confirmed extensive vitreous infiltration and optic nerve involvement. Histopathological analysis postmodified exenteration confirmed retinoblastoma. The patient clinically tolerated a modified exenteration and the first two of six planned cycles of systemic chemotherapy (vincristine, etoposide, and carboplatin). Unfortunately, she died during the second month of follow-up. The recurrence was characterized by progressive worsening of systemic symptoms and preauricular lymphadenopathy, likely indicating metastatic spread.

**Conclusion:** This case highlights the aggressive progression of advanced retinoblastoma and the consequences of delayed presentation in resource-limited settings. Although the patient demonstrated an initial positive response, clinically tolerating modified exenteration and two cycles of systemic chemotherapy, she rapidly succumbed to the disease. This underscores the critical need for early diagnosis, prompt referral, and improved access to specialized care to enhance outcomes in similar contexts.

## 1. Introduction

Retinoblastoma (RB) is recognized as the most prevalent primary intraocular malignancy in children, originating from the retinal embryonic cells [1, 2]. Early diagnosis plays a critical role in enhancing treatment success rates [1, 3]. Over the last 20 years, developed nations have made substantial progress in RB management, focusing not only on patient survival but also on the preservation of eyes and visual functions [2, 4]. In contrast, developing countries, including Nigeria, continue to experience high RB-related mortality, highlighting significant disparities in awareness, healthcare access, timely diagnosis, and treatment outcomes [1, 5–7]. Given the potential hereditary nature of RB, targeted genetic counseling and family screening may be considered in selecting high-risk cases such as those involving bilateral disease or early-onset presentations to facilitate early detection and guide clinical decision-making. While population-wide genetic screening is neither feasible nor justified, especially in low-resource settings, focused genetic evaluation in these specific contexts remains a valuable adjunct to clinical care. This report discusses a rare presentation of RB in a 9-year-old girl in Nigeria, treated at the ECWA Eye Hospital, a specialized tertiary facility committed to delivering comprehensive eye care [1].

## 2. Case Presentation

**Figure 1:**
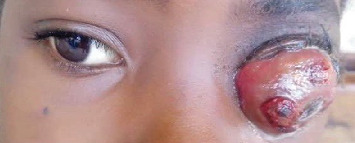
Initial clinical presentation of the patient at the time of evaluation at our center, showing a large, fungating ocular mass with axial proptosis, characteristic of advanced retinoblastoma.

A 9-year-old girl was referred to the ECWA Eye Hospital in Kano, Nigeria, with a 1-year history of persistent redness, tearing, pain, and progressive visual loss in her left eye. She was first evaluated at our facility on October 17, 2023. Approximately 4 months prior to presentation, the patient developed increasing swelling and protrusion of the left eyeball (Figure 1). Initial management at a local clinic involved the use of topical antibiotics and corticosteroids; however, no imaging or biopsy was conducted at that time. Her condition did not improve, and the family subsequently sought help from traditional healers before eventually presenting to our facility.

The patient is the second of three children in a nonconsanguineous family. Her father is a farmer, and her mother is a homemaker. There was no known family history of similar ocular conditions. On examination, the patient appeared chronically ill, with moderate pallor and peripheral lymphadenopathy involving the left preauricular, cervical, and submandibular lymph nodes. The presence of multiple enlarged lymph nodes raised concern for possible metastatic spread; however, no biopsy or histological confirmation was obtained to differentiate reactive from malignant causes, representing a limitation of the report.

Ophthalmic evaluation revealed a visual acuity of 6/6 in the right eye, while the left eye had no light perception. The left eye exhibited axial proptosis and a large, fungating, ulcerative ocular mass measuring 6 cm in both vertical and horizontal diameters. It was associated with purulent discharge, chemosis, and an indurated, opaque cornea (Figure 1). The right eye appeared unaffected on clinical examination, including slit-lamp and fundus assessments.

**Figure 2:**
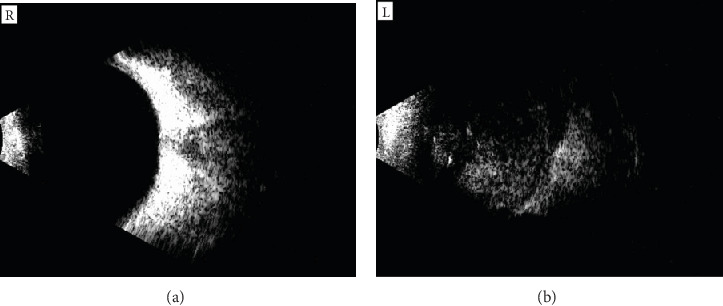
Ultrasound B-scan findings. (a) Normal appearance of the right eye. (b) Left eye demonstrating extensive vitreous infiltration by a solid mass, characterized by hyperechoic lesions and isolated calcified areas.

An initial diagnosis of left optic nerve glioma was considered, with a differential of rhabdomyosarcoma. A comprehensive diagnostic workup was conducted, including full blood count with white blood cell differential, hematocrit (36.4%), urea, creatinine, electrolytes, and liver function tests—all within normal limits. Orbital imaging was also performed. A B-scan ultrasonography of the left eye showed dense vitreous infiltration by a solid, hyperechoic mass containing isolated calcified areas (Figure 2b), while the right eye appeared normal (Figure 2a).

**Figure 3:**
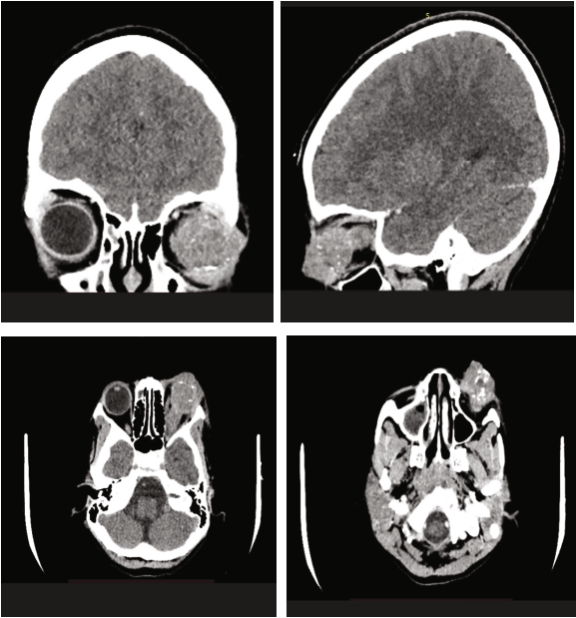
Computed tomography (CT) scan of the orbit and brain. A lobulated hyperdense mass involving the left globe and optic nerve with loss of globe contour, optic nerve thickening, and multifocal intraocular calcifications. The right orbit and extraocular muscles appear normal.

A computed tomography (CT) scan of the orbit and brain (Figure 3) revealed a lobulated soft tissue opacity in the anterior orbit on the left side. The lesion was predominantly hyperdense, involving the left globe and optic nerve, causing proptosis and a loss of the globe's spherical contour. There was marked thickening of the optic nerve and mild widening of the ipsilateral superior orbital fissure. Multifocal calcifications were noted within the globe, with poor visualization of the lens and vitreous chamber. The extraocular muscles and right orbit were normal.

**Figure 4:**
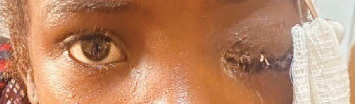
Postoperative appearance following modified exenteration of the left eye, showing the surgical site in the early recovery phase.

Following detailed counseling, the patient underwent a modified exenteration of the left globe and orbit. She remained clinically stable postoperatively but developed anemia (hematocrit 22%), which required a red blood cell transfusion. Once stabilized, she was prepared for systemic chemotherapy. A postoperative image is shown in Figure 4. The excised tissue was submitted for histopathological examination.

**Figure 5:**
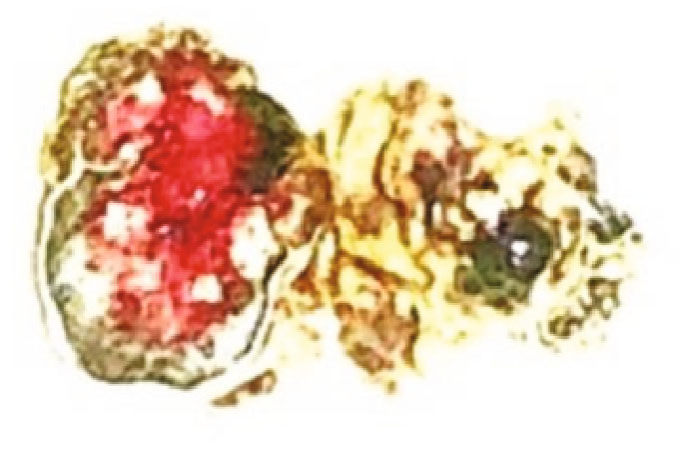
Macroscopic features of the excised orbito-ocular mass. The intraocular tumor measures 4 × 3 × 3 cm and extends through the cornea. The associated orbital mass measures 3 cm in diameter, displaying grayish–white to yellowish cut surfaces.

Gross pathological assessment (Figure 5) revealed an orbito-ocular mass measuring 4 × 3 × 3 cm and weighing 20 g. Sectioning showed the tumor occupying the intraocular space and extending anteriorly through the cornea. A separate orbital mass measuring 3 cm in diameter and weighing 12 g was also noted, displaying a grayish–white to yellowish cut surface.

**Figure 6:**
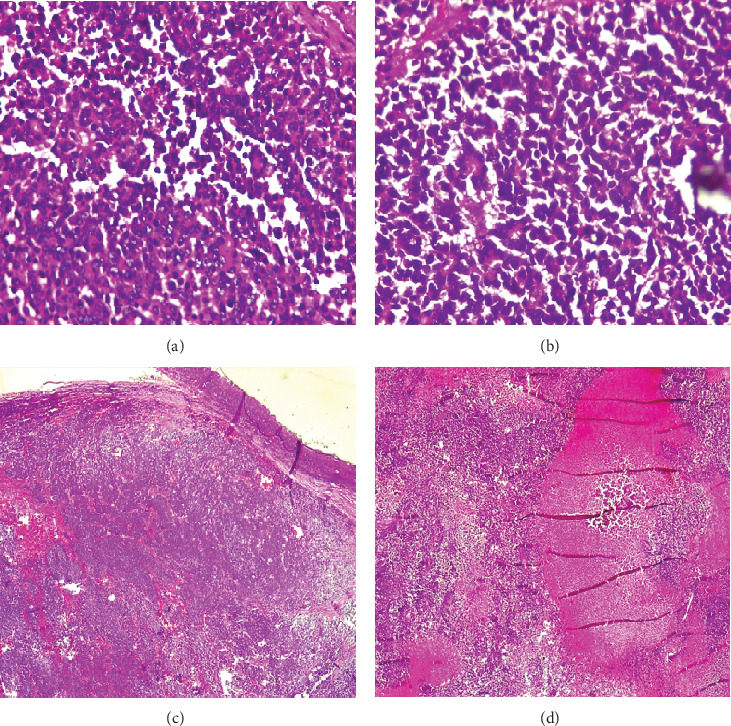
Histopathological micrographs of the excised tumor (H&E staining, magnification ×400). (a) Diffuse sheets of small, round blue cells. (b) Tumor cells interspersed with rosette formations. (c) Solid sheets of tumor cells within areas of necrosis. (d) Tumor infiltration of the optic nerve and periorbital fat.

Microscopic examination (Figure 6) revealed diffuse sheets of malignant, mitotically active small round blue cells (Figure 6a), areas of rosette formation (Figure 6b), and necrosis (Figure 6c). Tumor invasion through the sclera and cornea was confirmed. Sections of the orbital mass showed optic nerve expansion and periorbital fat infiltration by malignant cells (Figure 6d). No histochemical or immunohistochemical staining was conducted due to laboratory resource limitations.

Following a second pathologic opinion, the diagnosis of RB was confirmed. The family consented to a six-cycle chemotherapy regimen consisting of intravenous vincristine (0.05 mg/kg), etoposide (12 mg/kg), and carboplatin (28 mg/kg). The patient tolerated the first cycle well and was discharged after 24 h of observation. She returned after 28 days and again tolerated the second cycle without complications. Although there was no evidence of tumor regression, the patient remained clinically well throughout the first two cycles of chemotherapy.

During the second month of follow-up, the patient presented with worsening systemic symptoms, including progressive fatigue, weight loss, and enlargement of previously noted lymphadenopathy. There were no signs of neutropenia or active infection at that time. However, the clinical deterioration was strongly suggestive of disease progression and possible metastasis. Unfortunately, the patient died before the third chemotherapy cycle could be administered.

## 3. Discussion

This case illustrates the persistent challenges associated with the diagnosis and management of RB in low-resource settings, where delayed presentation remains a major barrier to successful outcomes [8–10]. While RB typically presents before the age of 5, cases in older children are not uncommon, particularly in sporadic, nonhereditary forms of the disease [2, 11, 12]. In such cases, the absence of a family history is typical and should not preclude timely diagnosis or clinical suspicion when progressive ocular symptoms are observed.

The clinical course in this case reflects trends documented in global epidemiologic studies. The Global Retinoblastoma Study Group reported that children in low-income countries present at a significantly higher median age (30.5 months) compared to those in high-income countries (14.1 months), with a markedly higher incidence of extraocular disease (49.1% vs. 1.5%) and correspondingly poorer outcomes [6]. Although Nigeria is classified as a lower-middle-income country, similar patterns of delayed presentation and advanced disease at diagnosis have been consistently reported across sub-Saharan Africa and other resource-constrained settings [5, 7–9]. These disparities reflect not only delayed diagnosis but also systemic limitations in awareness, screening, and timely referral within overstretched healthcare systems.

Our patient presented with an advanced unilateral tumor following a year-long progression of symptoms. Despite undergoing modified exenteration and initiating systemic chemotherapy with vincristine, etoposide, and carboplatin, the disease progressed rapidly, likely due to early metastatic spread. While these interventions were well tolerated and appropriate at the time of presentation, they were insufficient to reverse the course of an already advanced malignancy. This highlights the limited impact of aggressive therapy when applied too late in the disease trajectory [13, 14].

Improving outcomes for children with RB in developing nations will require a shift in focus toward early detection and public health intervention. A study conducted in Central America demonstrated that structured awareness campaigns, targeted training of healthcare providers, and decentralized screening initiatives can significantly reduce the age at diagnosis and decrease the incidence of extraocular RB [15]. Survival gains are most reliably achieved through timely identification and referral, rather than through escalation of care at advanced stages of disease [1, 6, 16].

This case also exemplifies the typical presentation of unilateral, sporadic RB in older children, with the contralateral eye remaining unaffected, as confirmed by both clinical examination and imaging. Histopathological evaluation revealed diffuse sheets of small round blue cells, rosette formation, areas of necrosis, and infiltration of the optic nerve and periorbital fat, consistent with advanced disease and high metastatic potential (Figure 6).

This case underscores the urgent need for coordinated national and regional public health strategies to promote early recognition and diagnosis of pediatric eye cancers. Strengthening education for caregivers and frontline health workers, improving access to eye care services, and establishing streamlined referral pathways are critical steps toward reducing late-stage presentations [15, 17]. Collaborative efforts between ophthalmology units, oncology centers, and public health agencies will be key to improving survival and long-term quality of life for children with RB in resource-constrained settings. Ultimately, while multidisciplinary care remains an essential component of treatment, its success depends largely on the timing of intervention [13, 18]. In environments where delayed diagnosis is common, sustainable progress hinges on systems that facilitate earlier access to expert care.

## 4. Conclusion

This case underscores the devastating impact of delayed diagnosis in pediatric RB and the limitations of initiating treatment at an advanced stage. Despite appropriate surgical and chemotherapeutic intervention following presentation, the patient's rapid decline and death highlight the urgency of early detection. Improving outcomes in such settings requires a shift in focus from late-stage intervention to timely diagnosis through strengthened public awareness, healthcare provider education, and accessible referral systems. Sustainable progress will depend on coordinated efforts to enhance early recognition and prompt access to specialized care, particularly in resource-constrained environments.

## Data Availability

The data that support the findings of this study are available from the corresponding authors upon reasonable request.
